# Effects of automated smartphone mobile recovery support and telephone continuing care in the treatment of alcohol use disorder: study protocol for a randomized controlled trial

**DOI:** 10.1186/s13063-018-2466-1

**Published:** 2018-01-30

**Authors:** James R. McKay, David H. Gustafson, Megan Ivey, Fiona McTavish, Klaren Pe-Romashko, Brenda Curtis, David A. Oslin, Daniel Polsky, Andrew Quanbeck, Kevin G. Lynch

**Affiliations:** 1 Center on Continuum of Care in Addictions, Perelman School of Medicine, University of Pennsylvania, Philadelphia VAMC, Philadelphia, PA 19104 USA; 2 Center for Health Enhancement System Studies, University of Wisconsin-Madison, Madison, WI 53706 USA; 3 Center for the Study of Addictions, Perelman School of Medicine, University of Pennsylvania, Philadelphia VAMC, Philadelphia, PA 19104 USA; 4 Leonard Davis Institute of Health Economics and Perelman School of Medicine, University of Pennsylvania, Philadelphia, PA 19104 USA; 5 Department of Family Medicine & Community Health, and Center for Health Enhancement Systems Studies, University of Wisconsin-Madison, Madison, WI 53706 USA; 6 Center on Continuum of Care in Addictions, Perelman School of Medicine, University of Pennsylvania, Philadelphia, PA 19104 USA

**Keywords:** Continuing care, Telephone counseling, A-CHESS, Smartphone, Automated recovery support, Mobile health, Alcohol use disorder, Alcohol use outcomes, Drug use outcomes, Intervention costs, Cost-effectiveness

## Abstract

**Background:**

New smartphone communication technology provides a novel way to provide personalized continuing care support following alcohol treatment. One such system is the Addiction version of the Comprehensive Health Enhancement Support System (A-CHESS), which provides a range of automated functions that support patients. A-CHESS improved drinking outcomes over standard continuing care when provided to patients leaving inpatient treatment. Effective continuing care can also be delivered via telephone calls with a counselor. Telephone Monitoring and Counseling (TMC) has demonstrated efficacy in two randomized trials with alcohol-dependent patients. A-CHESS and TMC have complementary strengths. A-CHESS provides automated 24/7 recovery support services and frequent assessment of symptoms and status, but does not involve regular contact with a counselor. TMC provides regular and sustained contact with the same counselor, but no ongoing support between calls. The future of continuing care for alcohol use disorders is likely to involve automated mobile technology and counselor contact, but little is known about how best to integrate these services.

**Methods/Design:**

To address this question, the study will feature a 2 × 2 design (A-CHESS for 12 months [yes/no] × TMC for 12 months [yes/no]), in which 280 alcohol-dependent patients in intensive outpatient programs (IOPs) will be randomized to one of the four conditions and followed for 18 months. We will determine whether adding TMC to A-CHESS produces fewer heavy drinking days than TMC or A-CHESS alone and test for TMC and A-CHESS main effects. We will determine the costs of each of the four conditions and the incremental cost-effectiveness of the three active conditions. Analyses will also examine secondary outcomes, including a biological measure of alcohol use, and hypothesized moderation and mediation effects.

**Discussion:**

The results of the study will yield important information on improving patient alcohol use outcomes by integrating mobile automated recovery support and counselor contact.

**Trial registration:**

ClinicalTrials.gov, NCT02681406. Registered on 2 September 2016.

**Electronic supplementary material:**

The online version of this article (10.1186/s13063-018-2466-1) contains supplementary material, which is available to authorized users.

## Background

Research on the etiology and course of substance use disorders makes a strong case for extended treatment [1]. Individuals who enter treatment often struggle with a number of factors that are slow to change or do not change at all and place them at heightened risk for relapse for considerable lengths of time [2]. These include genetic factors, interpersonal problems, co-occurring psychiatric disorders, employment problems, and various neurocognitive conditions [3–9]. Moreover, most positive factors associated with recovery, such as the development of supportive social networks, interests, and passions that reinforce abstinence, improved coping responses, employment, and other activities that provide a sense of worth and self-esteem, are slow to change and require ongoing support to prevent deterioration [1, 10, 11].

These findings may explain why treatments derived from an acute care model are of limited effectiveness in the long-term management of alcohol dependence. Specifically, vulnerability to relapse remains relatively high for significant periods after standard treatment protocols of 3–6 months have ended [12, 13]. Better management requires longer periods of continued contact with the patient [1, 14–16] to address flagging motivation, increased craving, diminished participation in self/mutual help, limitations in neurocognitive function, continued biological vulnerability to stress, and various other problems that arise.

Research has supported the effectiveness of extended continuing care. Reviews [17] found that positive effects were more likely when the continuing care intervention was at least 12 months long and employed active efforts to reach and retain patients. Examples of effective counselor delivered continuing care include behavioral marital therapy [18], home visits from a nurse [19], telephone contacts [20], recovery check-ups [12], extended contingency management [21], case management [22], extended monitoring in primary care [23], and extended integrated treatment models [24].

### Limitations in current approaches to continuing care

Although continuing care has been shown to be effective in a meta-analytic review [25], current approaches have four significant limitations. First, even when treatments are delivered with high fidelity to manuals, there is often considerable between-subject variability in outcomes and notable within-participant variability over time [26]. This can present a major challenge for extended models of care, which are often not flexible enough to provide rapid responses when patients do not respond to the intervention or deteriorate suddenly. Second, some relapse vulnerability factors can change rapidly—over periods as short as a few hours [2, 27]. A continuing care intervention in which data on relapse risks are obtained only during treatment sessions cannot be responsive to sudden shifts in risk level between sessions. This reduces the degree to which continuing care can be proactive or that timely information regarding increases in relapse risk can be communicated to peers and other sources of recovery support.

Third, patients are often urged to contact their counselors if they experience increases in relapse triggers in between regularly scheduled sessions. However, counselors may be working with other patients when the calls are made and are typically available only during regular clinic hours. Therefore, there are many hours during the week when it is not possible for a patient to speak to a counselor. Again, this limits the ability of continuing care to proactively address relapse risks and limit the severity of relapses when they occur. Finally, patients are urged to call peers in recovery and other supports when they feel at risk for relapse. However, patients may not have the necessary information when they most need it or may not be able to reach their contact person.

### Role for new communication technology in continuing care

New information and communication technologies (ICTs) [28, 29] may add to the efficacy of continuing care by addressing the limitations of these models and may yield cost savings [30]. Efficacy studies of ICTs in chronic disease self-management are promising [31–34]. People with addictions tend to view ICTs favorably [35] and they acknowledge more drug use and psychiatric symptoms online than in interviews [36]. Interactive voice response [37] has been associated with reductions in alcohol use [38]. ICTs boost motivation in health domains where social support is key to positive outcomes [39–41]. A review [42] found positive outcomes in 29 of 32 randomized trials of personal computer and single service (e.g. texting) cell phones for managing many different chronic diseases (e.g. addiction, pain, depression, cancer, diabetes, heart disease).

New mobile phone communication technology provides a way to bridge periods between continuing care sessions. It provides a personalized recovery support system during the evenings and on weekends when “live” professional counselors are unavailable [43–47]. A recent review summarized findings from studies in which mobile phones were used to enhance psychotherapy for a range of behavioral disorders [48]. In the studies that did calculate effects, the magnitude of effects favoring the mobile phone interventions was in the moderate-to-large range (d = 0.40–1.15). The authors concluded that more effective phone-based adjunctive interventions featured: (1) better integration of the telephone technology with psychotherapy; (2) mobile telephone protocols that clearly adhered to and supported the goals of the psychotherapy; and (3) face-to-face introductions to the program [48]. Recent studies not included in this review provide further evidence for the feasibility of using mobile phones as a component in therapy for adolescents [49] and in borderline personality disorder [50]. An important challenge for the alcohol treatment field is to determine how best to integrate new automated mobile recovery support technology and counselor- or therapist-delivered continuing care.

### Smartphone-based mobile health recovery support

For 25 years, David Gustafson and colleagues at the University of Wisconsin have developed and tested ICTs to improve health behaviors, quality of life, and access to care using an evolving needs-based platform called the Comprehensive Health Enhancement Support System (CHESS) for patients and family caregivers [51–53].

Addiction CHESS (A-CHESS) is a smartphone-based adaptation of CHESS that provides automated recovery support to individuals with substance use disorders. A-CHESS is consistent with self-determination theory and cognitive behavioral relapse prevention. Self-determination theory posits that satisfying three fundamental needs contributes to adaptive functioning: perceived competence; a feeling of relatedness (feeling connected to others); and autonomous motivation (feeling internally motivated and not coerced in one’s actions [54]). A-CHESS offers easy access anytime and anywhere to ten services tailored to meet patient needs. Services come in text and audio-video formats and include the following:

- *Social Relatedness services*: one-touch links to family, others in recovery, and discussion groups, via phone, email, and text messaging; GPS driven alerts to social supports
- *Coping Competence services*: regular assessment of risk and protective factors [55] to aid the patient in self-monitoring and inform others about the patient’s status, tailored information on coping, relaxation training and games to divert attention from craving, and Healthy Events Calendar
- *Autonomous Motivation services*: inspirational thoughts of the day, tailored support services linked to people who support their recovery, cognitive reframing that replaces negative thinking with opportunities for improvement, as well as monitoring and encouragements for sobriety

The A-CHESS system is ideally suited to address the four primary limitations in continuing care outlined earlier. Daily assessments of patient’s abstinence confidence, ongoing GPS monitoring, and “panic button” functions provide access to near real-time data that are not available from weekly or bi-monthly therapeutic contacts, which directly addresses heterogeneity of response and lack of between session information on patient status. The other features, including links to supporters and peers and tailored tools and information, provide more rapid access to social support and other recovery supports during periods when counselors are not available.

In a controlled trial, alcohol-dependent patients (*n* = 349) who had completed residential treatment were randomized to receive adjunctive A-CHESS for eight months or standard continuing care only. The participants continued to use the A-CHESS system at a high rate through the eight-month period during which it was provided. At eight months, 70% of participants were using A-CHESS at least weekly, compared to 92% at one month. Overall, participants used the system on 40% of days they had access to it. Patients receiving A-CHESS reported 49% fewer days of risky drinking in the prior 30 days at the four-, eight-, and 12-month follow-ups (mean of 1.39 days in A-CHESS vs 2.75 days in treatment as usual [TAU], *p* = 0.003), compared to those in TAU. Rates of alcohol abstinence within the prior 30 days were higher in A-CHESS than in TAU at the eight-month follow-up (78% vs 67%) and 12-month follow-up (79% vs 66%) (*p* < 0.04) [56].

### Telephone-based continuing care

McKay et al. developed a flexible, patient-centered approach to the long-term management of substance use disorders, Telephone Monitoring and Counseling (TMC) [57]. The theoretical basis of TMC comes from Stress and Coping Theory [58, 59], which emphasizes the identification of high-risk situations, increasing self-efficacy, and improving coping strategies; and Social Control Theory [60], which stresses monitoring, structure, and goal direction. These goals are also consistent with the primary goals of the Chronic Care Model, as described by Wagner et al. [16], which include support for patient self-management, links to community resources, interventions to increase self-confidence and skill levels, a focus on goal setting, and identification of barriers to achieving goals and methods to overcome such barriers.

Prior studies of TMC have found: (1) for alcohol- and/or cocaine-dependent graduates of four-week intensive outpatient programs (IOPs), TMC produces better drug and alcohol use outcomes than traditional group counseling continuing care [61]; (2) augmenting more lengthy IOPs (i.e. 3–4 months) with TMC produces significantly better alcohol use outcomes than IOP only [20], but mixed outcomes with cocaine-dependent patients [61, 62]; (3) TMC effects are mediated by increased attendance at self-help meetings, increased commitment to abstinence, and increased self-efficacy [63]; (4) TMC is more effective for poorer prognosis patients, such as those who fail to achieve abstinence early in treatment, have poor social support, or low motivation, and for women [62, 64, 65]; and (5) TMC is cost-effective [66, 67].

### Rationale for study design

We believe that the future of continuing care and disease management for alcohol use disorders will involve a combination of new automated communication/social networking technologies and contact with actual counselors. The 2 × 2 study design in this study will make it possible to determine whether there is a synergistic effect of TMC and A-CHESS, such that the combination is significantly better than either intervention alone. This design also allows for replication of main effects found in prior studies and the first direct head-to-head comparison of TMC and A-CHESS.

### Economic evaluation of TMC and A-CHESS

In the design of effective treatment programs for clients suffering from alcohol dependence, it is not sufficient to just determine the most effective program because treatment typically occurs in resource-constrained environments. If these treatments were found to be equally effective, the one most likely to be adopted would be the less expensive one. If the combined treatment was more effective than any single treatment, the question is whether the additional effectiveness is worth the extra expense. In this study we will use incremental cost-effectiveness analysis (CEA) to quantify the tradeoff between the additional effectiveness of the continuing care interventions. Cost-effectiveness is assessed by comparing the incremental cost-effectiveness ratio (ICER) to what decision-makers are willing to pay for an additional unit of effect. If the ICER for one strategy is below this willingness-to-pay threshold, then the strategy should be considered potentially cost-effective.

**Fig. 1:**
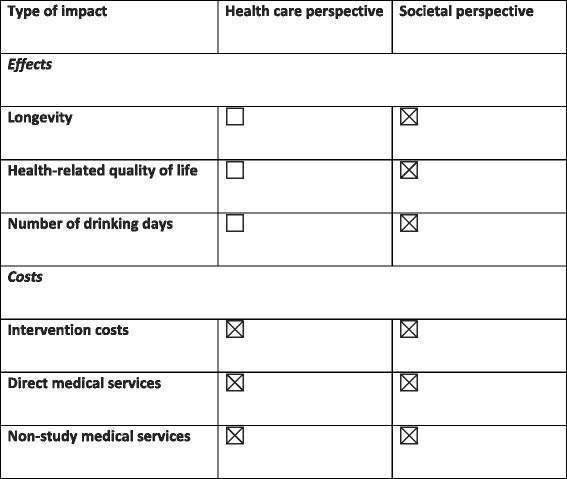
Impact inventory template. Form used to cost out treatment interventions (adapted from Sanders et al. [69])

By assessing cost-effectiveness, we will be able to provide data relevant to determining how to best maximize resource allocation and minimize financial barriers to adoption of effective and cost-effective interventions [68]. In this case, the alternatives would be either single continuing care treatment vs usual care and the combined continuing care treatments vs either alone. This is a particularly significant addition to this study because TMC and A-CHESS are relatively inexpensive additions to outpatient treatment and are very inexpensive substitutes for returns to inpatient treatment or intensive outpatient treatment. It is therefore possible that these interventions will be cost effective in the current study, as was the case with TMC in prior studies, and such an analysis could be an important contributor to broad uptake of these innovative interventions. We will conduct the CEA from two perspectives: the societal perspective as well as the healthcare system perspective. As recommended in recently updated recommendations on conducting CEA in healthcare, we will also include an “impact inventory” to show how costs and effects accrue to different healthcare stakeholders [69]. A template showing the structure of an impact inventory is illustrated in Fig. 1.

### Potential moderator effects

Patients with greater substance use severity, poor social support, low motivation, and multiple prior treatments have poorer treatment outcomes [64, 65], and will therefore benefit to a greater degree from TMC and A-CHESS. It is conceivable that the combination of TMC and A-CHESS will also be of greater benefit to patients with prior treatment experience and greater alcohol use severity before or in the first three weeks of IOP and in those with less social support for recovery and lower motivation after the first three weeks of IOP. In secondary, exploratory analyses, we will examine whether these factors moderate TMC and A-CHESS main effects and the comparison of TMC + A-CHESS to the other treatment conditions.

### Identifying factors that mediate hypothesized main effects

The A-CHESS system is designed to increase three key constructs within self-determination theory [54]: coping competence; social support; and motivation (9). In secondary analyses, we will assess variables that represent these constructs and analyze whether they mediate effects favoring TMC or A-CHESS or their combination. The mediating effect of self-efficacy will also be examined in the main effect comparison of TMC, as it has been shown to mediate telephone continuing care effects in our prior work [63].

## Methods/Design

### Aim

The primary objective of the study is to test for main effects for TMC and A-CHESS and the combination of TMC + A-CHESS on the primary alcohol use outcome in IOP participants with moderate-to-severe alcohol use disorder. Economic analyses will examine the cost-effectiveness of each intervention from two perspectives: that of society, and that of the healthcare system. Secondary objectives are to test three hypotheses concerning secondary outcomes, moderating effects, and mediation effects.

### Design

The proposed 2 × 2 randomized trial employs an experimental, prospective design, in which 280 individuals with current, moderate-to-severe alcohol use disorder will be randomly assigned into four conditions and followed for 18 months. The follow-ups will be at three, six, nine, 12, and 18 months post baseline.

### Setting

Participants are being recruited from two Philadelphia area IOPs. These IOPs provide traditional, 12-step oriented treatment, delivered through 9 h of (primarily) group counseling per week. Patients who do not drop out of treatment in the first two weeks typically are retained for 3–4 months in the program.

### Participants

The participants are patients in treatment in two Philadelphia inner-city IOPs, with current moderate-to-severe alcohol use disorder and aged 18–75 years. We anticipate the study sample to average about 40 years of age; 75% of the participants will be African-American, 21% will be White, and 4% will be other minorities. Approximately 10% will be Hispanic and 40% will be female. About one-third will be employed and all will have a relatively stable residence.

To be eligible for participation, patients must: (1) have a DSM-V diagnosis of current moderate-to-severe alcohol use disorder; (2) have completed three weeks of IOP; (3) be aged 18–75 years; (4) have no current psychotic disorder or dementia severe enough to prevent participation in treatment; (5) have no acute medical problem requiring immediate inpatient treatment; (6) not be on methadone or in other forms of substance abuse treatment, other than IOP; and (7) be willing to participate in a randomized clinical trial. Finally, participants will (8) be able to provide the name, verified telephone number, and address of at least two contacts willing to provide locator information on the patient during follow-up; and (9) be functionally literate and have sufficient visual ability to read the smartphone. Other substance use disorders will not exclude IOP patients from participation, provided they have current moderate-to-severe alcohol use disorder. IOP patients who are also receiving treatment for co-occurring psychiatric or medical problems will not be excluded either, as long as they meet other inclusion/exclusion criteria.

### Participant recruitment

When patients enter IOP, they are told about the study by IOP staff. Patients who express interest in participating are referred to a research technician on site. The research technician explains the study, obtains an initial informed consent for screening, and administers a brief instrument that collects demographic data and eligibility screening information. Patients are then given an appointment for the baseline assessment to be conducted three weeks later. If the baseline is not scheduled at the time of screening, the research technician schedules the appointment at a later date via telephone, or with permission from the eligible participant, via SMS text message. Patients who choose not to participate in the study continue to receive treatment as usual.

We also display study recruitment flyers and leaflets at the recruitment sites with the site director’s approval. Patients who become interested in the study from seeing our flyer or leaflet can call a research technician to complete a phone screening interview. The patient provides verbal consent to complete the phone screen. If the patient is determined to be eligible, they are scheduled for a baseline appointment. At the initial baseline assessment, patients who agree to participate sign a second informed consent, after they have successfully passed an informed consent quiz. Patients who meet criteria for participation then complete the remainder of the baseline assessments and are randomly assigned to one of the four treatment conditions. However, we placed the first seven participants into the TMC + A-CHESS treatment condition to pilot our procedures and ensure smooth delivery of this combined intervention before randomizing other participants.

### Baseline interviews

**Fig. 2:**
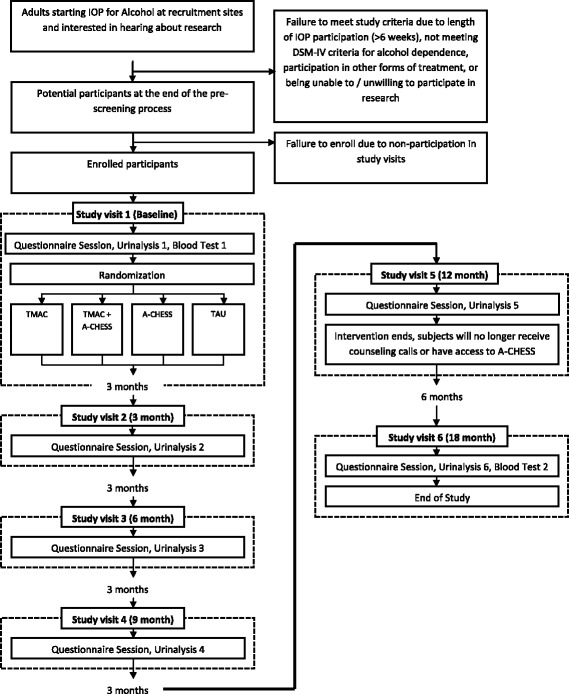
Study *flow chart*

Baseline assessments are conducted in weeks 4–6 of the IOP. The baseline visit includes consenting and gathering contact information, assessments, a urine specimen, and a blood draw. All procedures are for research purposes only and are not part of the participants’ clinical chart at their IOP. At the completion of the baseline visit after final determination of eligibility has been completed, the participants are randomized and compensated $40.00 regardless of which arm they are in. A flow chart of study procedures is presented in Fig. 2.

### Assessment instruments

**Fig. 3:**
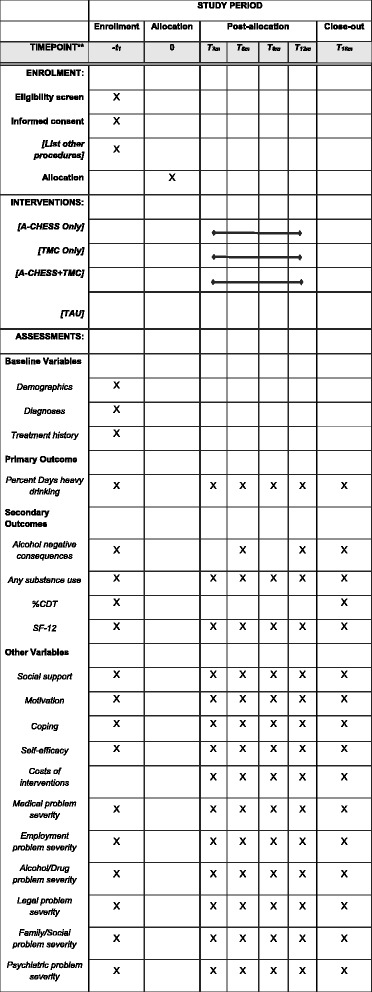
Study assessment points and instruments. SPIRIT figure

Assessment points and instruments are presented in Fig. 3. The following assessment instruments were used in the study. The Addiction Severity Index (ASI) [70] was used to assess demographics, alcohol and drug use, treatment history, and problem severity levels in the areas of alcohol and drug use, medical status, psychiatric status, legal status, employment status, and family/social support. The Time-Line Follow-Back (TLFB) [71] was used to assess frequency of alcohol and heavy alcohol use. The Structured Clinical Interview for DSM-IV (SCID) [72] was used to determine alcohol and drug use disorder and psychiatric DSM-IV diagnoses. The Short Inventory of Problems (SIP) [73] was used to assess negative consequences of alcohol use. Percent carbohydrate deficient transferrin (%CDT) [74] provided a biological measure of heavy alcohol use over the past several months. Level of functioning was assessed with the Short Form Survey (SF-12) [75]. Processes of Change [76] was used to assess coping. Social support for recovery was assessed with the Important People Interview (IPI) [77]. Self-efficacy was assessed with the alcohol version of the  Drug Taking Confidence Questionnaire (DTCQ) [78]. Commitment to abstinence was assessed with Thoughts About Abstinence [79]. Costs of treatment interventions was determined by the DATCAP [80] and EQ-5D [81].

### Assignment of interventions and blinding

Participants are randomized to either: (1) A-CHESS Only for 12 months; (2) TMC Only for 12 months; (3) A-CHESS + TMC for 12 months; or (4) TAU. The allocation sequence is provided by computer-generated random numbers, blocking on groups of 16. The randomization is stratified by sex and co-occurring drug use, by site. The assignments are placed in sealed envelopes by research technicians who are not involved with the study. Due to the nature of the interventions and staffing for the study, it was not possible to blind research technicians to study condition.

### Treatment interventions

#### A-CHESS Only

After participants are randomized to this condition, they meet with a research technician to receive their smartphone. The research technician enters the following information into the A-CHESS system: participant demographics; healthy events of interest to participant; high-risk locations; and key relapse triggers. Protocols for initiating contact with other people are discussed and programmed into the smartphone (i.e. high-risk locations for GPS monitoring, protocols for what happens when panic button is pushed, etc.). A password is selected by the participant and used to protect the phone. Participants are also trained to use the A-CHESS system during this session. Follow-up training is also available through brief video tutorials for each A-CHESS service and at research follow-up visits. Participants can use the recovery support functions of A-CHESS whenever they wish. Following seven days of inactivity, the system sends a message to the participant and to a member of the research staff monitoring A-CHESS utilization, who encourage A-CHESS use via text messages. Technical support for A-CHESS operation is available via phone.

Each day, A-CHESS contacts participants to obtain information on confidence for maintaining abstinence. Once per week, participants are also prompted to complete a brief ten-item assessment of risk and protective factors. Risk factors include sleep difficulties, emotional distress, urges to drink/craving, tempting situations, and interpersonal problems. Protective factors include abstinence self-efficacy, involvement in Alcoholics Anonymous (AA) or other mutual support groups, spirituality, social support, and engagement in productive activities. These items were taken from the Brief Addiction Monitor (BAM), which is now widely used within the VA to monitor patient progress. A-CHESS combines that information with data from prior assessments to predict relapse in the coming week. If a participant exceeds a threshold, an alert is sent to A-CHESS staff and the participant is encouraged via text messages to seek additional support (see below).

A-CHESS provides links to relevant resources. For participants with low abstinence confidence or worrisome scores on the risk or protective items of the progress assessment, A-CHESS automatically provides suggestions of relevant coping skills. Participants receive tailored feedback that acknowledges their alcohol or drug use over the past week and provides recommendations of relevant coping skills for addressing risky behaviors based on their BAM responses.

A-CHESS also offers relaxation exercises, games for distraction, connections to online peer support, links to a healthy events newsletter, suggestions for diversionary activities, and contact with the participant’s support system. Participants have access to discussion groups populated by other participants in the study, via online bulletin board, or text messaging. Guidelines for appropriate use of these formats are stressed while patients use A-CHESS. Any mention of use of a phone for illegal purposes in A-CHESS discussion group results in the phone being turned off. Mobile software allows participants to text their location to pre-approved friends, family, and peers so that they can respond to requests for help.

Additional A-CHESS features include access to audio and written information on addiction, web links, GPS-driven information on local self-help meetings and treatment services, inspirational messages, and reminders via texting to take medication and attend appointments. Participants may also use the smartphone to access the web and make telephone calls so that they do not have to carry two phones.

Participants receiving A-CHESS Only do not have regular contact with a continuing care counselor. Rather, when participants report low confidence or other concerning data in the weekly assessment, the system sends a message to the patient and to a member of Dr. Gustafson’s staff monitoring A-CHESS utilization, who encourages more active use of recovery supports via text messages. We provide up to one replacement smartphone to participants when they report a phone lost or stolen. For those who also lose the second smartphone, we offer to load the A-CHESS program onto a smartphone that they obtain on their own. We do replace broken or defective smartphones, as long as the participant brings it in to our research offices.

#### Telephone Monitoring and Counseling Only

Participants have one face-to-face session with the counselor who will provide TMC to them, to enable the counselor to develop initial rapport, explain the intervention, establish goals for the treatment, and provide a copy of a workbook to the participant. Telephone calls occur weekly for the first month, twice monthly for the next three months, monthly for months 4–7, and every other month for months 8–12 (i.e. 16 possible calls). Each call is initiated either by the counselor or the participant, depending on which method will yield the greatest likelihood of a successful connection in that case.

Each telephone call is 15–30 min in duration. At the beginning of the call, the participant completes the brief Progress Assessment. These data are analyzed in real time with a computer program developed in our prior studies, which yields summary scores for risk factors, protective factors, and the ratio of risk to protective factors. Participant and counselor go over the 1–2 goals that the participant is working on and objectives that need to be accomplished to reach each goal. Problems that were identified in the Progress Assessment are addressed and coping behaviors for any anticipated upcoming high-risk situations are identified and rehearsed. In addition, reinforcement of participant strengths and positive behaviors, and further encouragement for involvement in pro-recovery lifestyle activities, are provided.

In our prior work, TMC was delivered by counselors who were part of the research team and were employees of the University of Pennsylvania. However, the original design of this study called for TMC to be provided by counselors who work at the IOPs, to better approximate how the intervention would actually be delivered in most programs if it were to be disseminated. At the start of the study, we therefore trained several counselors at each IOP to provide TMC and TMC + A-CHESS. Due to high rates of counselor turnover at the two IOPs, we have had periods where no IOP counselors were available to take on new participants. Therefore, we have used our University of Pennsylvania counseling staff to provide the study interventions in these cases.

For participants randomized to the TMC Only condition who do not have reliable access to a telephone, a cell phone with unlimited talk is offered to allow the participant to engage in telephone counseling intervention and contact study personnel.

#### TMC and A-CHESS (TMC + A-CHESS)

Each participant has one face-to-face session (60–75 min) with the IOP counselor or University of Pennsylvania counselor who provides the telephone intervention to him/her, to enable the counselor to develop an initial rapport with the participant, explain the protocol, and establish initial goals for the treatment. Programming of the participant’s smartphone, protecting it with a password, and orientation to A-CHESS are done by research technicians in a separate meeting with participants, shortly after randomization has occurred. Subsequent telephone calls occur on the same schedule as in the TMC Only condition (see above).

As described above, participants are contacted daily by A-CHESS and queried about their confidence in remaining abstinent for the day and weekly to administer the brief progress assessment. When participants report worrisome information in the prompts (see A-CHESS description above), alerts are sent directly to counselors as long as the participant approves. A graph with current BAM scores and scores from the past few assessments can be seen by the counselor in the dashboard. In addition, participants are able to activate their smartphones at any time and complete additional BAM assessments if they wish to do so. These procedures provide counselors with timely information on relapse risk.

The counselor dashboard helps the counselors to quickly identify participants who may be at high risk for relapse. Counselors can see changes in participant scores, A-CHESS use, and if they had relapsed. The counselors can intervene by calling participants or texting them in A-CHESS. When a counselor logs into the counselor dashboard, he/she sees “red pins” generated when there is a significant decline in participants’ weekly BAM scores, inactivity in A-CHESS, or drugs or alcohol use in the past week.

#### Treatment as usual

Participants in this condition receive standard care in the IOP, plus access to weekly step-down standard outpatient care if they complete IOP and wish to continue. They do not receive A-CHESS or TMC. As is the case in virtually all public treatment programs in the USA, all treatments at our two recruitment sites are based on 12-step principles and are delivered almost entirely through group counseling sessions.

### Research follow-ups

Regardless of study condition and whether participants are active in the intervention, all participants are followed for research visits at three, six, nine, 12, and 18 months after baseline. At the completion of each follow up, participants are paid $50. To achieve high follow-up rates, we focus on patient education and motivation, collection of extensive and verified locator information, between-assessments contacts via the mail and telephone, confirmation of follow-up appointments before follow-up date, and standardized tracking procedures.

### Data entry and monitoring

Most of the study data will be entered directly into databases as it is collected, via the Penn Center’s web-based data entry system. These databases are password- and firewall-protected and do not contain the participant’s name or any other identifying information. Data forms that are not amenable to web-based data entry will be transported to the PI’s data entry center and will be entered in the computer independently by two teams of trained data entry staff; discrepancies will be corrected by a supervisor, based on source documents. The quality of the data will be monitored once per month.

### Adverse events

Serious adverse events (SAEs) will be systematically assessed at each clinic visit. Any SAE, whether related to study intervention or not, will be reported to the Institutional Review Board (IRB) and the National Institute on Alcohol Abuse Alcoholism (NIAAA; the funder). The initial SAE report will be followed by submission of a completed SAE report to both institutions. In the event that a patient either withdraws from the study or the investigator decides to discontinue a patient due to SAE, the patient will be monitored by the investigator via ongoing status assessment until either a resolution is reached (i.e. the problem requiring hospitalization has resolved or stabilized with no further changes expected), the SAE is determined to be clearly unrelated to the study intervention, or the SAE results in death. Outcome of SAEs will be periodically reported to NIAAA. A summary of the SAEs that occurred during the previous year will be included in the annual progress report to NIAAA.

### Primary outcome variable(s)

The primary outcome measure is percent days of heavy alcohol use (i.e. ≥ 5 drinks/day for men, ≥ 4 drinks/day for women) within each follow-up period. The primary endpoint is therefore percent days heavy alcohol use during months 13–18 (i.e. the final follow-up period). Studies have consistently supported the reliability and validity of the TLFB with alcohol-dependent individuals. Frequency of heavy alcohol use was selected because alcohol-related problems are correlated with the frequency of heavy drinking days. This outcome is also sensitive to reductions in problematic or high-risk use, which are particularly important in a disease management model.

### Secondary outcome variable(s)

Four secondary outcomes will also be examined: alcohol use-related negative consequence (SIP); any substance use within a follow-up period (yes/no, as determined by TLFB, ASI, and urine drug screens); carbohydrate deficient transferrin (%dCDT); and quality of life (as assessed with the SF-12). The first two measures were selected to provide a fuller picture of overall substance use and severity of drinking consequences. Although the study interventions are primarily focused on reducing alcohol use, reductions in other drug use and negative consequences are clearly desirable and clinically important. CDT was included to provide a biological measure of heavy drinking, to corroborate results obtained with the self-report TLFB. Finally, the quality of life measure was included to obtain a more global, overall health outcome. Data are also obtained on the frequency, timing, and total time in each A-CHESS service, including when and what service is accessed. These data also include the timing, content, and completeness of weekly and daily assessments.

Data on retention in IOP, outpatient SUD treatment sessions, and treatment costs are obtained through treatment program records, participant self-report, the EQ-5D, and the DATCAP.

### Data analytic approach

The responses for the primary hypotheses comprise continuous measurements over the three-, six-, nine-, 12-, and 18-month follow-up points. Our main analyses will compare the interventions using mixed effects linear regression models for frequency (percent days) of heavy alcohol use and other continuous outcomes, and mixed effects logistic regression models for dichotomous outcomes. Given the small number of time points, we will regard time as a categorical variable, although we may simplify the model if smoother (polynomial or spline, for example) time trends appear adequate for model fit.

#### Intervention effects

The main explanatory variables will be binary factors indicating TMC and A-CHESS. The models will also include TMC × A-CHESS, TMC × time, and A-CHESS × time interactions, as necessary for model fit based on AIC/BIC comparisons. The estimated regression coefficients for the intervention indicators and their interaction, and for group by time interactions, will address our primary hypotheses on overall A-CHESS and TMC effects; a contrast statement on the coefficients will address the comparison between the group receiving both A-CHESS and TMC and the combined groups receiving A-CHESS Only or TMC Only. The secondary comparison of A-CHESS Only vs TMC Only will also be addressed using contrasts of the fitted model coefficients. Further comparisons, including the comparison of each of A-CHESS Only and TMC Only against TAU, will be addressed in a similar way.

The design permits a full description of the effects of the two interventions. If a significant A-CHESS × TMC interaction is obtained, we will report the separate estimates and confidence intervals of the effect of either intervention across the level of the other. Even if there is no significant interaction, a model including that term will allow us to estimate the magnitude of the benefit of adding a second intervention relative to either one alone. Finally, if the non-significant interaction is dropped from the model, the separate TMC and A-CHESS terms will estimate and test the (possibly time varying) effects of each intervention.

Our primary analyses will rely on randomization for group balance (along with the urn process), while baseline measures on which the groups differ will be examined for inclusion as covariates in supplementary analyses. Based on analyses of similar data, we expect that a random intercept model, possibly with an auto-regressive repeated measures structure, should provide a good fit to the covariance structure of the repeated measures. The secondary outcomes (negative consequences of drinking, alcohol and drug abstinence, %dCDT, and quality of life) will be analyzed using generalized linear mixed effects models appropriate for the response distributions, following the same approach described above for the primary heavy drinking outcome.

#### Moderator analyses

We will perform a series of moderator analyses, using a set of baseline participant characteristics: frequency of heavy drinking before treatment; prior treatments for alcoholism; and alcohol use, poor social support, and low motivation in the first three weeks of IOP (i.e. during IOP but before randomization). For each analysis, we will extend the mixed effects models described above for the primary hypotheses to include the main effect of the moderator, and its interaction with the binary factors for intervention groups, and the group by time interactions. If inclusion of the interaction terms for a given moderator yields significantly better model fit, this provides evidence for moderation. Data plots and the estimated regression coefficients for the intervention, the moderator, and their interaction will explain the nature and direction of the moderating effect.

#### Mediation analyses

Analyses will be conducted to examine the potential mediating effects of increases in coping, social support, readiness to change, and self-efficacy on the primary heavy drinking outcome. We will first perform a series of analyses to assess whether improvements in coping, social support, and readiness to change between baseline and the six-month follow-up mediate the treatment effects of A-CHESS and of TMC at the 12-month follow-up. Similar analyses will be performed to determine if improvement between baseline and 12 months mediate treatment effects at 18 months. These are cross-sectional analyses, as the response of interest is being considered at a single time point. We will supplement these analyses with causal mediation models [82, 83]. These causal models can be considered as sensitivity analyses for the first set of models, as they do not explicitly require the absence of confounding between treatment effect and mediator level. We will also supplement these cross-sectional models by longitudinal mediation models [84], utilizing the repeated measures on both the main response and the mediators themselves. Similar procedures will be used to examine these potential mediating effects in the comparison of the group receiving TMC + A-CHESS vs the combined groups receiving either A-CHESS Only or TMC Only.

#### Multiple comparisons and the control of type I error

We are comparing two intervention main effects on one primary outcome and also comparing the group receiving both interventions to the combined groups receiving only one of the interventions. Therefore, we selected an alpha level of 0.0167 to control the overall probability of type 1 error at 5%. For our secondary outcomes, and other analyses, our goal is to generate rather than to confirm hypotheses, so no control of type I error will be performed.

#### Economic analyses

The primary economic outcome to be examined is the ICER, which will inform an adoption decision from both: (1) the societal perspective; and (2) the perspective of the healthcare system. The primary ICER will be the incremental costs per fewer days drinking (the clinical measure of effectiveness) and the secondary ICER will be incremental costs per increased quality-adjusted life year (QALY) (the economic measure of effectiveness). We will use an 18-month time horizon for our primary analysis. Costs are estimated using resource costing. For this method, one multiplies the dollar value of each economic resource (i.e. price weight) by the count of units of that resource for each study participant. The University of Wisconsin team has adapted a data collection instrument originally developed for economic assessment of addiction treatment programs and used it for cost data collection on two recent NIDA-funded studies: one assessing the value of quality improvements in addiction treatment [85], the other studying dissemination of A-CHESS in primary care (1 R01 DA034279-01). The instrument will assess costs related to delivery of the two interventions (including training, development, supervision of counselors, etc.) and the marginal costs of using the interventions in combination. The units of medical resource use come from clinical forms as well as the non-study medical services form for resources used outside of the scope of the intervention. Summation of these products provides an estimate of total medical costs for each participant.

Proper estimation of cost-effectiveness requires statistical modeling of the costs and QALYs using generalized linear model (GLM) and inverse probability weighting (IPW) to account for missing data [86] and integrating these models into the ratio and the uncertainty around that ratio by applying bootstrapping methods [86]. We will test whether the cost-effectiveness ratios are lower than a range of acceptable maximum cost-effectiveness (CE) ratios. There is no single standard acceptable threshold, but $50,000 and $100,000 per QALY are often used by convention in CEAs conducted from a societal perspective [87]. From the perspective of the healthcare system, cost considerations are likely of prime concern. We will examine the extent to which costs of the interventions are offset by reductions in direct medical costs and use of non-study medical services (e.g. emergency room visits, hospitalizations). Sensitivity analyses will be performed to assess the sensitivity of our estimates of costs and cost-effectiveness to the assumptions made regarding some of the values that will be used in the analysis. We will consider how cost-effectiveness may change with: (1) different costs of inputs for the interventions; (2) different methods to handle attrition of cost data; and (3) different maximum CE ratios.

### Statistical power

To control the overall probability of type 1 error, we set an alpha level of 1.67% (see above). The analyses will use mixed effects models, where the focus is on the transformed (log or square-root) percent days heavy drinking per assessment period. We base power estimates on a combination of the methods of Hedeker et al. [88] and Stroup [89]. Based on prior studies, we expect an overall loss to dropout of about 20% by the 18-month time point and a within-subject correlation of 0.35. With these assumptions and an alpha level of 1.67%, the sample of size 280 provides 83% power for an effect of d = 0.29 for the main effect of either intervention with a 22% dropout. The design is balanced with respect to A-CHESS and TMC, so these main effect power estimates are valid whether there is an A-CHESS × TMC interaction or not. With the same assumptions, we have 80% power for an effect of d = 0.34 between TMC + A-CHESS vs A-CHESS Only and TMC Only. With power to find effect sizes in the d = 0.29–0.34 range, we will have adequate power to detect clinically meaningful differences between the treatment conditions.

## Discussion

This study will generate important information on the impact of combining counselor-delivered continuing care via the telephone (TMC) with an automated, multifunction mobile health recovery support system delivered via smartphone (A-CHESS). The analyses will examine main effects for TMC and A-CHESS and the TMC × A-CHESS interaction on the primary alcohol use outcome (percent days heavy drinking) and four secondary outcomes: alcohol use-related negative consequences; any substance use within a follow-up period; a biological measure of heavy drinking (carbohydrate deficient transferrin or %dCDT); and quality of life. Participants are being recruited from two IOPs, after completing three weeks of treatment. The continuing care treatment interventions will be provided for 12 months and participants will complete follow-ups at three, six, nine, 12, and 18 months post baseline.

The projected sample size of 280 provides sufficient power to find moderate size main and interaction effects, with adjustments to alpha for the number of primary comparisons examined. The analyses will examine the cost-effectiveness of the three experimental conditions relative to each other and treatment as usual. Hypothesized moderator and mediator effects will also be tested. Therefore, the study will provide data on the efficacy of two evidence-based continuing care interventions separately and in a combined and integrated format, intervention costs and cost-effectiveness, mechanisms of action, and moderators.

Although the study design is generally strong, it has several limitations. If significant treatment condition effects are obtained for TMC and A-CHESS vs TAU, we will not be able to determine if the effects are specific to these interventions or simply indicate that additional counseling services or the provision of a smartphone are helpful. Due to turnover in the IOPs and problems finding counselors who were willing and able to participate in the study, we have had to use our research counselors rather than IOP counselors to provide telephone continuing care to approximately half of the participants to date. We will attempt to address this in the analyses by comparing results in the TMC and TMC + A-Chess conditions for IOP vs UPenn counselors. Finally, the follow-up period may not be long enough to detect potential economic benefits of the continuing care interventions, as both may drive up treatment costs in the short run (see Additional file 1).

### Trial status

Protocol version: 1

Date recruitment began: 27 April 2015

Approximate date recruitment will be completed: 1 August 2018

### Resource sharing plans

At the conclusion of the study and after the principal findings have been analyzed and disseminated, Drs. McKay and Gustafson will entertain requests for data sharing from outside investigators. In general, the PIs and co-investigators are committed to the widest possible dissemination of findings but also feel that the study should be represented and interpreted appropriately. Therefore, any third party that requests and is given access to the data will also be obligated to communicate to Drs. McKay and Gustafson any manuscript generated from this study at least one month before submitting it for publication. Before any data sharing, all data will be carefully examined to make sure that the privacy of study participants is protected through thorough de-identification procedures.

### Interim analyses

Blind interim analyses of the data will be conducted at two points when 50% and 75% of the sample has been accrued. If the results show statistically overwhelming significant differences between groups, the study will be stopped (or one of the conditions stopped).

## Additional file

Additional file 1: SPIRIT 2013 Checklist: Recommended items to address in a clinical trial protocol and related documents*. (DOC 122 kb)

## Abbreviations

%dCDT: Carbohydrate deficient transferrin
A-CHESS: Addiction-Comprehensive Health Enhancement Support System
CE: Cost-effectiveness
GLM: Generalized linear model
ICTs: Information and communication technologies
IOP: Intensive outpatient program
IPW: Inverse probability weighting
QALYs: Quality-adjusted life years
SF-12: Short Form 12 Health Survey
SIP: Short Index of Problems
TAU: Treatment as usual
TLFB: Time-Line Follow-Back
TMC: Telephone Monitoring and Counseling

## References

[1] McKay JR (2009). Treating substance use disorders with adaptive continuing care.

[2] McKay JR, Franklin TR, Patapis N, Lynch KG (2006). Conceptual, methodological, and analytical issues in the study of relapse. Clin Psychol Rev.

[3] Kalivas PW (2007). Neurobiology of cocaine addiction: Implications for new pharmacotherapy. Am J Addict.

[4] Koob GF, Volkow ND (2010). Neurocircuitry of addiction. Neuropsychopharmacology.

[5] Miller WR, Westerberg VS, Harris RJ, Tonigan JS (1996). What predicts relapse? Prospective testing of antecedent models. Addiction.

[6] Moos RH, Finney JW, Cronkite RC (1990). Alcoholism treatment: context, process, and outcome.

[7] Weisner C, Delucchi K, Matzger H, Schmidt L (2003). The role of community services and informal support on five-year drinking trajectories of alcohol dependent and problem drinkers. J Stud Alcohol.

[8] Kranzler R, Edenberg J (2010). Pharmacogenetics of alcohol and alcohol dependence treatment. Curr Pharm Des.

[9] Kreek M, Nielsen D, Butelman E, LaForge K (2005). Genetic influences on impulsivity, risk taking, stress responsivity and vulnerability to drug abuse and addiction. Nat Neurosci.

[10] Moos RH, Moos BS (2007). Treated and untreated alcohol-use disorders: Course and predictors of remission and relapse. Eval Rev.

[11] Orford J, Hodgson R, Copello A, John B, Smith M, Black R (2006). The clients’ perspective on change during treatment for an alcohol problem: Qualitative analysis of follow-up interviews in the UK Alcohol Treatment Trial. Addiction.

[12] Dennis M, Scott C, Funk R (2003). An experimental evaluation of recovery management checkups (RMC) for people with chronic substance use disorders. Eval Program Plann.

[13] McLellan A, Lewis D, O’Brien C, Kleber H (2000). Drug dependence, a chronic medical illness: implications for treatment, insurance, and outcomes evaluation. J Am Med Assoc.

[14] Dennis ML, Scott CK (2007). Managing addiction as a chronic condition. Addict Sci Clin Pract.

[15] McLellan AT, McKay JR, Forman R, Cacciola J, Kemp J (2005). Reconsidering the evaluation of addiction treatment: From retrospective follow-up to concurrent recovery monitoring. Addiction.

[16] Wagner EH, Austin BT, Davis C, Hindmarsh M, Schaefer J, Bonomi A (2001). Improving chronic illness care: Translating evidence into action. Health Aff (Millwood).

[17] McKay JR (2009). Continuing care research: What we have learned and where we are going. J Subst Abuse Treat.

[18] O’Farrell TJ, Choquette KA, Cutter HS (1998). Couples relapse prevention sessions after behavioral marital therapy for male alcoholics: Outcomes during the three years after starting treatment. J Stud Alcohol.

[19] Patterson DG, Macpherson J, Brady NM (1997). Community psychiatric nurse aftercare for alcoholics: A five-year follow-up study. Addiction.

[20] McKay JR, Van Horn DH, Oslin DW, Lynch KG, Ivey M, Ward K (2010). A randomized trial of extended telephone-based continuing care for alcohol dependence: Within-treatment substance use outcomes. J Consult Clin Psychol.

[21] Silverman K, Robles E, Mudric T, Bigelow GE, Stitzer ML (2004). A randomized trial of long-term reinforcement of cocaine abstinence in methadone-maintained patients who inject drugs. J Consult Clin Psychol.

[22] Morgenstern J, Blanchard KA, McCrady BS, McVeigh KH, Morgan TJ, Pandina RJ (2006). Effectiveness of intensive case management for substance-dependent women receiving temporary assistance for needy families. Am J Public Health.

[23] Kristenson H, Österling A, Nilsson J, Lindgärde F (2002). Prevention of alcohol related deaths in middle aged heavy drinkers. Alcohol Clin Exp Res.

[24] Willenbring ML, Olson DH (1999). A randomized trial of integrated outpatient treatment for medically ill alcoholic men. Arch Intern Med.

[25] Blodgett JC, Maisel NC, Fuh IL, Wilbourne PL, Finney JW (2014). How effective is continuing care for substance use disorders? A meta-analytic review. J Subst Abus Treat.

[26] Morgenstern J, McKay JR (2007). Rethinking the paradigms that inform behavioral treatment research for substance use disorders. Addiction.

[27] Shiffman S, Waters AJ (2004). Negative affect and smoking lapses: A prospective analysis. J Consult Clin Psychol.

[28] Hilbert M, Lopez P (2011). The world’s technological capacity to store, communicate, and compute information. Science.

[29] Pentland A. Reality mining of mobile communications: Toward a new deal on data. In: Dutta S, Mia I, editors. The global information technology report 2008–2009. Geneva: World Economic Forum; 2009. p. 75–80.

[30] Popovici I, French MT, McKay JR (2008). Economic evaluation of continuing care interventions in the treatment of substance abuse: recommendations for future research. Eval Rev.

[31] Bewick B, Trusier K, Barkham M, Hill A, Cahill J, Mulhern B (2008). The effectiveness of web-based interventions designed to decrease alcohol consumption: A systematic review. Prev Med.

[32] Burns MN, Begale M, Duffecy J, Gergle D, Karr CJ, Giangrande E (2011). Harnessing context sensing to develop a mobile intervention for depression. J Med Internet Res.

[33] Granholm E, BenZeev D, Link PC, Bradshaw KR, Holden JL (2012). Mobile assessment and treatment for schizophrenia (MATS): A pilot trial of an interactive textmessaging intervention for medication adherence, socialization, and auditory hallucinations. Schizophr Bull.

[34] Reid SC, Kauer SD, Hearps SJ (2011). A mobile phone application for the assessment and management of youth mental health problems in primary care: a randomised controlled trial. BMC Fam Pract.

[35] Cunningham JA, Wild TC, Walsh GW (1999). Interest in self-help materials in a general population sample of drinkers. Alcohol Educ Prev Policy.

[36] Rosen CS, Henson BR, Finney JW, Moos RH (2000). Consistency of self-administered and interview-based: Addiction Severity Index composite scores. Addiction.

[37] Simpson TL, Kivlahan DR, Bush KR, McFall ME (2005). Telephone self-monitoring among alcohol use disorder patients in early recovery: A randomized study of feasibility and measurement reactivity. Drug Alcohol Depend.

[38] Helzer J, Badger G, Rose GL, Mongeon J, Searles JS (2002). Decline in alcohol consumption during two years of daily reporting. J Stud Alcohol.

[39] Bickmore TW, Picard RW (2005). Establishing and maintaining long-term human-computer relationships. ACM Trans Comput Human Interact.

[40] Bickmore TW, Pfeifer LM, Jack BW. Taking the time to care: Empowering low health literacy hospital patients with virtual nurse agents, Paper presented at the International Conference on Human Factors in Computing Systems, New York, NY. 2009.

[41] Bickmore T, Gruber A (2010). Relational agents in clinical psychiatry. Harv Rev Psychiatry.

[42] Gustafson DH, Boyle MG, Shaw BR, Isham A, McTavish F, Richards S (2011). An e-health solution for people with alcohol problems. Alcohol Res Health.

[43] Boschen MJ, Casey LM (2008). The use of mobile telephones as adjuncts to cognitive behavioral psychotherapy. Prof Psychol Res Pract.

[44] Cohn AM, Hunter-Reel D, Hagman BT, Mitchell J (2011). Prompting behavior change from alcohol use through mobile technology: The future of ecological momentary assessment. Alcohol Clin Exp Res.

[45] Galloway GP, Didier R, Garrison K, Mendelson J (2008). Feasibility of ecological momentary assessment using cellular telephones in methamphetamine dependent subjects. Subst Abuse.

[46] McKay JR. The use of new communication technologies to evaluate and intervene in substance dependence. Curr Behav Neurosci Rep. 2015;2:23–29. 10.1007/s40473-014-0017-y PMC5844699 29527457

[47] Gustafson DH, Shaw BR, Isham A, Baker T, Boyle MG, Levy M (2011). Explicating an evidence-based, theoretically informed, mobile technology-based system to improve outcomes in people in recovery for alcohol dependence. Subst Use Misuse.

[48] Clough BA, Casey LM (2011). Technological adjuncts to enhance current psychotherapy practices: A review. Clin Psychol Rev.

[49] Matthews M, Doherty G. In the mood: Engaging teenagers in psychotherapy using mobile phones. Proceedings of the SIGCHI Conference on Human Factors in Computing Systems. Vancouver: ACM; 2011. p. 2947–2956.

[50] Rizvi SL, Dimeff LA, Skutch J, Carroll D, Linehan MM (2011). A pilot study of the DBT coach: An interactive mobile phone application for individuals with borderline personality disorder and substance use disorder. Behav Ther.

[51] Gustafson DH, Taylor J, Thompson S, Chesney P (1993). Assessing the needs of breast cancer patients and their families. J Qual Manag Healthc.

[52] Boberg EW, Gustafson DH, Hawkins RP, Offord KP, Koch C, Wen KY (2003). Assessing the unmet information, support and care delivery needs of men with prostate cancer. Patient Educ Couns.

[53] DuBenske LL, Wen KY, Gustafson DH, Guarnaccia CA, Cleary JF, Dinauer SK (2008). Caregivers differing needs across key experiences of the advanced cancer disease trajectory. Palliat Support Care.

[54] Deci EL, Ryan RM (2004). Handbook of self-determination research.

[55] Shiffman S, Stone A, Hufford M (2008). Ecological momentary assessment. Annu Rev Clin Psychol.

[56] Gustafson DH, McTavish FM, Chih M-Y, Atwood AK, Johnson RA, Boyle MG (2014). A smartphone application to support recovery from alcoholism: A randomized controlled trial. JAMA Psychiatry.

[57] McKay JR, Van Horn D, Morrison R (2010). Telephone continuing care therapy for adults.

[58] Folkman S, Lazarus RS, Kunkel-Schetter C, DeLongis A, Gruen RJ (1986). Dynamics of a stressful encounter: Cognitive appraisal, coping, and encounter outcomes. J Pers Soc Psychol.

[59] Kaplan HB, Kaplan HB (1996). Psychosocial stress from the perspective of self theory. Psychosocial stress: Perspectives on structure, theory, life course, and methods.

[60] Moos RH (2007). Theory-based active ingredients of effective treatments for substance use disorders. Drug Alcohol Depend.

[61] McKay JR, Lynch KG, Shepard DS, Pettinati HM (2005). The effectiveness of telephone-based continuing care for alcohol and cocaine dependence: 24-month outcomes. Arch Gen Psychiatry.

[62] McKay JR, Van Horn DHA, Lynch KG, Ivey M, Cary MS, Drapkin M (2013). An adaptive approach for identifying cocaine dependent patients who benefit from extended continuing care. J Consult Clin Psychol.

[63] Mensinger JL, Lynch KG, TenHave TR, McKay JR (2007). Mediators of telephone-based continuing care for alcohol and cocaine dependence. J Consult Clin Psychol.

[64] McKay JR, Van Horn DHA, Oslin D, Lynch KG, Ivey M, Drapkin ML (2011). Extended telephone-based continuing care for alcohol dependence: 24 month outcomes and subgroup analyses. Addiction.

[65] McKay JR, Van Horn DHA, Lynch KG, Ivey M, Cary MS, Drapkin M (2014). Who benefits from extended continuing care?. Addict Behav.

[66] McCollister K, Yang K, McKay JR (2016). Cost-effectiveness analysis of a continuing care intervention for cocaine-dependent adults. Drug Alcohol Depend.

[67] Shepard DS, Daley MC, Neuman MJ, Blaakman AP, McKay JR (2016). Telephone-based continuing care counseling in substance abuse treatment: Economic analysis of a randomized trial. Drug Alcohol Depend.

[68] Drummond M, Sculpher M, Torrance G, O’Brien B, Stoddart G (2005). Methods for the economic evaluation of health care programmes.

[69] Sanders GD, Neumann PJ, Basu A, Sculpher M, Brock DW, Feeney D (2016). Recommendations for conduct, methodological practices, and reporting of cost-effectiveness analyses: Second panel on cost-effectiveness in health and medicine. JAMA.

[70] McLellan A, Kushner H, Metzger D, Peters R, Smith I, Grissom GR (1992). The fifth edition of the Addiction Severity Index. J Subst Abus Treat.

[71] Sobell LC, Brown J, Leo GI, Sobell MB (1996). The reliability of the Alcohol Timeline Followback when administered by telephone and by computer. Drug Alcohol Depend.

[72] First MB, Spitzer RL, Gibbon M, Williams JBW (2002). Structured Clinical Interview for DSM-IV-TR Axis I Disorders, Research Version, Patient Edition. (SCID-I/P).

[73] Feinn R, Tennen H, Kranzler HR (2003). Psychometric properties of the short index of problems as a measure of recent alcohol-related problems. Alcohol Clin Exp Res.

[74] Helander A, Wielders JP, Jeppsson JO, Weykamp C, Siebelder C, Anton RF (2010). Toward standardization of carbohydrate-deficient transferrin (CDT) measurements: II. Performance of a laboratory network running the HPLC candidate reference measurement procedure and evaluation of a candidate reference material. Clin Chem Lab Med.

[75] Ware JE, Kosinski M, Keller SD (1996). A 12-Item Short-Form Health Survey: Construction of scales and preliminary tests of reliability and validity. Med Care.

[76] Litt MD, Kadden RM, Cooney NL, Kabela E (2003). Coping skills and treatment outcomes in cognitive-behavioral and interactional group therapy for alcoholism. J Consult Clin Psychol.

[77] Zywiak W, Neighbors C, Martin R, Johnson J, Eaton C, Rohsenow D (2009). The Important People Drug and Alcohol interview: Psychometric properties, predictive validity, and implications for treatment. J Subst Abus Treat.

[78] Annis H, Martin G (1985). Drug-taking confidence questionnaire (DTCQ).

[79] Hall SM, Havassy BE, Wasserman DA (1991). Effects of commitment to abstinence, positive moods, stress, and coping on relapse to cocaine use. J Consult Clin Psychol.

[80] French MT, Dunlap LJ, Zarkin GA, McGeary KA, McLellan AT (1997). A structured instrument for estimating the economic cost of drug abuse treatment: the drug abuse treatment cost analysis program (DATCAP). J Subst Abus Treat.

[81] The EuroQol Group (1990). EuroQol-a new facility for the measurement of health-related quality of life. Health Policy.

[82] TenHave TR, Joffe MM, Lynch KG, Brown GK, Maisto SA, Beck AT (2007). Causal mediation analyses with rank preserving models. Biometrics.

[83] Lynch KG, Cary MS, Gallop R, TenHave TR (2008). Causal mediation analyses for randomized trials. Health Serv Outcomes Res Methodol.

[84] MacKinnon DP (2008). Introduction to statistical mediation analysis.

[85] Gustafson DH, Quanbeck AR, Robinson JM, Ford JH, Pulvermacher A, French MT (2013). Which elements of improvement collaboratives are most effective? A cluster-randomized trial. Addiction.

[86] Glick HA, Doshi JA, Sonnad SS, Polsky D (2007). Economic evaluation in clinical trials.

[87] Hirth RA, Chernew ME, Miller E, Fendrick AM, Weissert WG (2000). Willingness to pay for a quality-adjusted life year: In search of a standard. Med Decis Mak.

[88] Hedeker D, Gibbons RD, Waternaux C (1999). Sample size estimation for longitudinal designs with attrition. J Educ Behav Stat.

[89] Stroup WW (1999). Mixed model procedures to assess power, precision, and sample size in the design of experiments.

